# Impact of combined ischemic preconditioning and remote ischemic perconditioning on ischemia-reperfusion injury after liver transplantation

**DOI:** 10.1038/s41598-018-36365-5

**Published:** 2018-12-19

**Authors:** Ding-yang Li, Wen-tao Liu, Guang-yi Wang, Xiao-ju Shi

**Affiliations:** 1 Department of Hepatobiliary & Pancreatic Surgery, The First Affiliated Hospital of Zhengzhou University, Zhengzhou, 450000 Henan Province China; 2 Department of Hepatobiliary & Pancreatic Surgery, The First Norman Bethune Hospital Affiliated to Jilin University, Changchun, 130021 Jilin Province China

## Abstract

Ischemic preconditioning (IPC) and remote ischemic perconditioning (RIPer) confer protective effects against liver ischemia-reperfusion injury (IRI), but data about RIPer applying in liver transplantation is lacking. The study aimed to evaluate whether the combination of IPC and RIPer provides reinforced protective effects. C57BL/6 mice (160 pairs) were allocated into four groups: control, subjected to liver transplantation only; IPC, donor hilar was clamped for 10 min followed by 15 min of reperfusion; RIPer, three cycles of occlusion (5 min) and opening (5 min) of femoral vascular bundle were performed before reperfusion; IPC + RIPer, donors and recipients were subjected to IPC and RIPer respectively. Liver tissues were obtained for histological evaluation, TUNEL staining, malondialdehyde assays, GSH-Px assays, and NF-κB p65 protein and Bcl-2/Bax mRNA analyses. Blood samples were used to evaluate ALT, AST, TNF-α, NOx levels and flow cytometry. We found that protective efficacy of RIPer is less than IPC in terms of ALT, TNF-α, GSH-Px and NOx at 2 h postoperation, but almost equivalent at 24 h and 72 h postoperation. Except for Suzuki scores, ALT, Bcl-2/Bax mRNA ratio, other indices showed that combined treatment brought enhanced attenuation in IRI, compared with single treatment, through additive effects on antioxidation, anti-apoptosis, modulation of microcirculation disturbance, and inhibition of innate immune response. This study suggested a combined strategy that could enhance protection against IRI in clinical liver transplantation, otherwise, provided a hint that RIPer’s mechanism might be partly or totally different from IPC in humoral pathway.

## Introduction

Although liver transplantation, as the only effective therapeutic method for end-stage liver disease, has been widely applied, unavoidable hepatic ischemia-reperfusion injury (IRI) is still challenging for clinicians because of the increased risk for hypohepatia, primary graft failure, allograft vasculopathy, acute rejection reactions, and damage to other major organs^1,2^. This problem needs to be addressed more pressingly with extension of the donor pool using marginal grafts followed by severer IRI^3,4^.

The protective effect of ischemic preconditioning (IPC) against IRI was first reported in 1986, which has been verified in various tissues and organs^5–7^. However, the potential traumatic risks from direct stress to the target organ and prolonged total ischemic time limits its clinical practicability. In comparison, remote ischemic perconditioning (RIPer) described by Schmidt in 2007 has stronger applicability and maneuverability, which confers protective effects through several brief ischemias followed by reperfusion in remote tissues or organs during the ischemic phase^8^.

It has been confirmed that IPC initiates an endogenous mechanism of resistance to liver injury by regulating energy metabolism, reducing the generation of free radicals, improving microcirculation disturbance, inhibiting inflammatory reactions, and inducing the release of endogenous protective factors^6,9^. The liver-protective mechanism of RIPer has not been fully elucidated because this method was developed recently and the related research has mostly focused on the heart, brain, and kidney^10–12^. RIPer is now thought to trigger a complex neurohumoral process that includes the release of blood-borne protective factors dependent on prior activation of sensory afferent nerves, the transfer of the protective signal through blood circulation, neural pathways, and/or systemic responses, which ultimately exert the protective effect in the target organ^13,14^. Recently, the co-dependence of the neuro/humoral pathways was proposed in a remote ischemic conditioning model of rat arterialized orthotopic liver transplantation^15^.

A number of animal and clinical studies have assessed the protective efficacy of IPC in liver transplantation and hepatectomy^16,17^. Some animal studies showed hepatic protection of RIPer from warm IRI^14,18^. The RIPer model in liver transplantation was first established in 2015 by Zheng SS using rats, subsequently identified the involvement of antioxidants, inhibition of immune responses, and phosphatidylinositide 3-kinase/Akt/endothelial nitric oxide synthase/nitric oxide pathways in protective mechanism^19,20^. Considering the condition that experimental studies about the effect of RIPer in liver transplantation are still severely lacking, here we chose a novel model of RIPer with mouse liver transplantation to assess the effect of combined IPC and RIPer. In addition, we compared the protective efficacies of the conditioning methods against IRI in terms of liver injury, cell apoptosis, oxidative stress, microcirculation, and inflammatory responses.

## Methods

### Animals and experimental design

Ten to fourteen-week-old male C57BL/6 mice (Vital River Laboratories, China) weighing 25–30 g were housed under specific pathogen-free conditions with a 12 h light/dark cycle. All mice were fasted without water deprivation for 12 h before liver transplantation. A total of 160 pairs of mice were randomly and equally assigned into four groups that received no treatment, IPC, RIPer, or IPC + RIPer. The animal experiment protocol was approved by the Jilin University Animal Care and Use Committee. All mice were treated in accordance with the National Institutes of Health guide for the care and use of Laboratory animals (NIH Publications No. 8023, revised 1978). Our preliminary experiment demonstrated that storage of the graft at 0–4 °C in Ringer’s solution for 3 h could lead to severe but reversible liver injury. Thus, we chose this duration as the cold ischemia time.

### Surgical procedures

Mouse orthotopic liver transplantation (MOLT) was performed using a surgical microscope (SZX 3.0, Olympus, Japan) under inhalation anesthesia with isoflurane. Detailed procedures have been described previously^21^.

**Figure 1:**
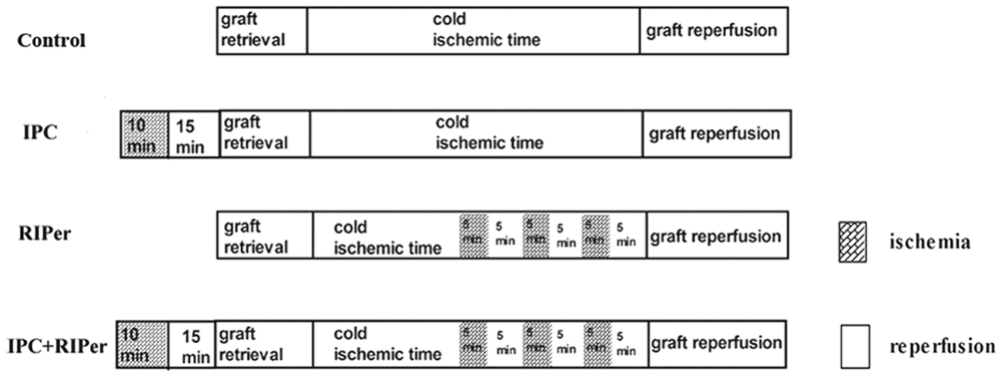
Protocol applied in each study group (n = 10).

Briefly, in control group, mice were subjected to MOLT only. In IPC group, the donor hepatic artery and portal vein were clamped with a microvascular clamp for 10 min of ischemia followed by 15 min of reperfusion before graft procurement as we described before^22^. In RIPer group, during the time from anesthetization to graft reperfusion, three cycles of occlusion (5 min) and opening (5 min) of the bilateral femoral vascular bundle with microvascular clamps were performed as described previously^23^. To ensure the homogeneity, we set the time that starting the second cycle of opening the limb vascular bundle at the onset of anheptic phase. In IPC + RIPer group, the donor and recipient were subjected to IPC and RIPer respectively (Fig. 1). To avoid the influence of surgical time, we opened the abdominal cavity at 28 minutes before graft retrieval for Control and RIPer group. In addition, we set the time length from recipient anesthetization to the onset of anhepatic phase as 15 minutes for all groups.

Recipients that died during anesthesia or from operational errors were excluded from analyses, then equivalent mice were added. In accordance with the random number table, ten mice in each group were chosen to be followed for one month. Each group was then randomly divided into three subgroups according to terminated time points including 2, 24, and 72 h after graft reperfusion. Blood was obtained via puncture of the abdominal vena cava to evaluate ALT, AST, TNF-α, NOx levels and for flow cytometric analysis. The middle lobe of liver was removed and fixed in 10% formaldehyde for histological evaluation and TUNEL staining. The remaining liver tissue was immediately snap frozen in liquid nitrogen and stored at −80 °C until used for malondialdehyde assays, GSH-Px assays, NF-κB p65 protein and Bcl-2/Bax mRNA analyses.

### Histological examination

Harvested livers were fixed in 10% formalin, embedded in paraffin, sectioned at 4 μm thicknesses, and stained with hematoxylin and eosin for general histopathological evaluation of IRI according to Suzuki’s criteria^24^.

Apoptosis of hepatocytes was detected by a Trans Detect *In Situ* Fluorescein TUNEL Cell Apoptosis Detection Kit (Beyotime, China). Only hepatocytes were included in analysis. The apoptotic index was determined as the percentage of apoptotic cells per hepatic cell population in five random fields at x400 magnification (IX51, Olympus).

### Serum assays

Serum ALT and AST were assayed with an automatic biochemistry analyzer (Vitros 350, Johnson & Johnson, USA). Serum TNF-α was measured by an enzyme-linked immunosorbent assay (#430906, Biolegend, USA). The serum total nitrite and nitrate (NOx) concentration was measured with a nitric oxide fluorometric assay kit (#K252, Biovision, USA).

### Flow cytometric analysis

White blood cells were isolated in blood using red blood cell lysis buffer (Beyotime, China). Rat anti-mouse CD11b-APC (# 17-0113-42, eBioscience, USA) and rat anti-mouse CD16/32-PE (#101308, Biolegend, USA) were used in analysis of innate immune responses. Rat immunoglobulin G2a was used as the isotype control (#553432, BD Pharmingen, USA). Labeled cells were analyzed using a FACSCalibur (BD Biosciences, USA).

### Molecular analysis of liver tissue

The supernatant was collected after centrifugation (12000 rpm for 10 min) of homogenates prepared using a tissue grinder. Malondialdehyde (MDA) and glutathione peroxidase (GSH-Px), common biomarkers of lipid peroxidation and anti-peroxidation, respectively, were assayed in the supernatant using colorimetric and ultraviolet chromatometry methods with a Lipid Peroxidation MDA Assay Kit (#S0131, Beyotime, China) and Cellular Glutathione Peroxidase Assay Kit (#S0056, Beyotime, China), respectively, according to the manufacturers’ protocols.

Total protein was extracted using mammalian protein extraction reagent (Boster, China). Protein concentrations were determined with a BCA protein quantitative kit (Boster, China). Rat anti-mouse NF-κB p65 monoclonal antibody (#sc-71675, Santa Cruz Biotechnology, USA) diluted at 1:500 was used with horseradish peroxidase-conjugated rabbit anti-rat immunoglobulin (#AR1170, Boster, China) diluted at 1:2000. Protein bands were detected using an ECL chemiluminescence detection system (Amercontrol Biosciences, USA) and gel imaging system (Chemi Doc XRS+, USA).

### Statistical analysis

Data are presented as means ± standard error of the mean. Differences between two dependent groups were evaluated with the paired Student t-test. Comparisons among multiple groups were performed with one-way analysis of variance followed by Bonferroni post-hoc tests. Animal survival analysis was performed using the Kaplan-Meier method and log-rank test. All p-values were two-tailed. A value of p < 0.05 was regarded as statistically significant.

## Results

### No effect of the conditioning methods on survival rate

Five mice were excluded from the study because of anastomotic bleeding (two mice), longer anhepatic phase (two mice), or laceration of common bile duct (one mouse), the total incidence of mortality during and immediately after performing surgical operation was 3.0% (5/165). Anhepatic and total cold ischemic times were 17.3 ± 1.4 min and 235.7 ± 18.3 min, respectively, and there was no significant difference among four groups (p = 0.12. Postoperative graft survival at 1 month in control, IPC, RIPer and IPC + RIPer group was 80%, 80%, 70%and 80%, respectively, and no difference in survival curves was observed (p > 0.05).

### Effect of the conditioning methods on liver injury

**Figure 2:**
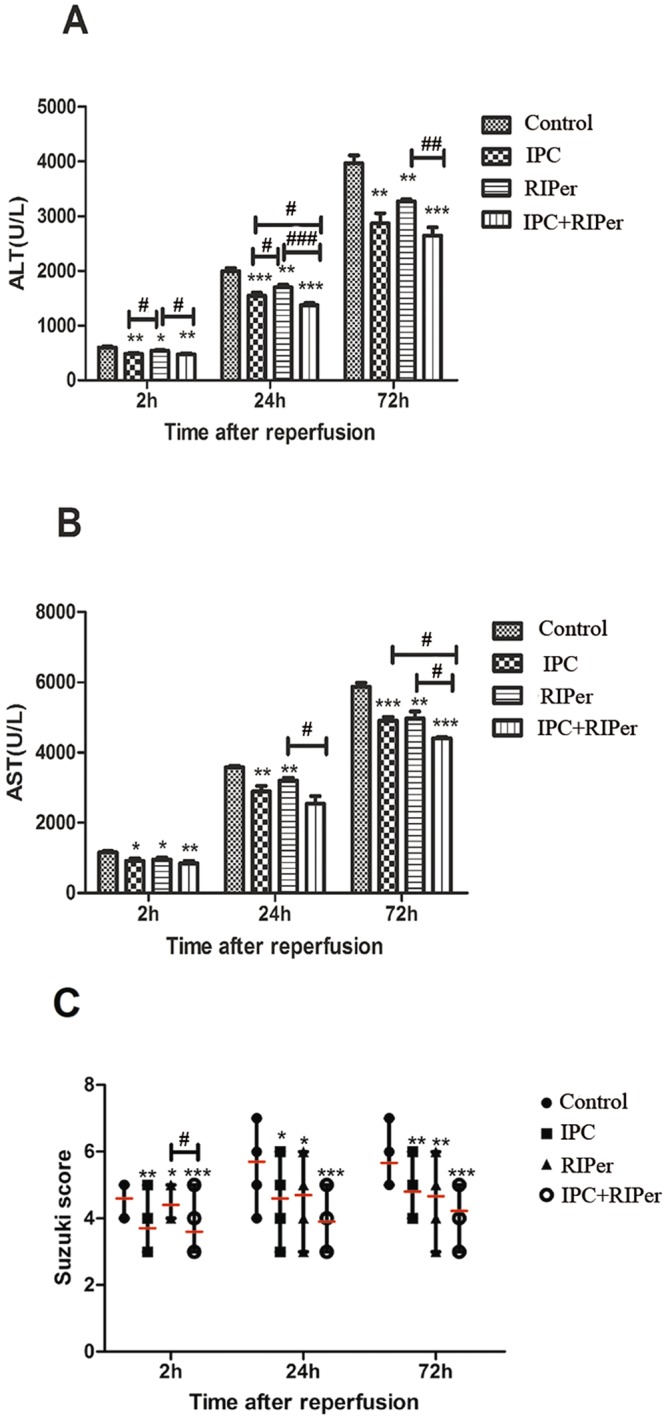
Effect of different maneuvers on the IRI of liver grafts. Serum ALT level (**A**), AST level (**B**) and Suzuki injury score (**C**) were detected at 2 h, 24 h and 72 h after liver transplantation, “-” indicated mean value. *p < 0.05; **p < 0.005; ***p < 0.0005 vs control group, paired Student t-test. ^#^p < 0.05; ^##^p < 0.005; ^###^p < 0.0005 vs different conditioning method, one-way ANOVA and Bonferroni post-hoc test.

Liver injury was evaluated by ALT, AST, and histopathology. The levels of ALT and AST in each group showed progressive tendencies. The ascending range in conditioning groups was smaller than that in control group. At 2 h and 24 h after reperfusion, ALT in IPC group was lower than that in RIPer group (p = 0.03). Both ALT at 24 h and AST at 72 h after reperfusion indicated enhanced attenuation of IRI in IPC + RIPer group (Fig. 2A,B).

Histological changes showed a gradually increased tendency of liver injury with time in all groups. At 2 h after reperfusion, we can find a large number of swelling hepatocytes and hepatic sinus endothelial cells, tiny spot of hepatic necrosis. At 24 h, vacuolar change of hepatocytes, hepatic necrosis in adjacent lobules and sinusoidal dilatation appeared. At 72 h, hepatic necrosis and vacuolar change became more severe, there was a slight decrease in blood stasis in hepatic sinusoid. Suzuki score in each conditioning group was lower than that in control group. There was no difference in IPC and RIPer groups. In addition, the score at 2 h demonstrated that addition of IPC improved the protective efficacy of RIPer alone (Fig. 2C).

### Effect of the conditioning methods on immune responses

Activation of innate immune system, accumulation of inflammatory cytokines, and regulation of signaling pathways are closely associated with liver injury. CD11b is expressed on the surface of myeloid cells, and coexpression with CD16/32 promotes adhesion and phagocytosis, indicating activation of myeloid cells. TNF-α, a cytokine produced by monocytes and macrophages in response to inflammatory stimuli. NF-κB p65, an eukaryotic transcription factor that could regulate a lot of molecules taking part in inflammatory reaction.

**Figure 3:**
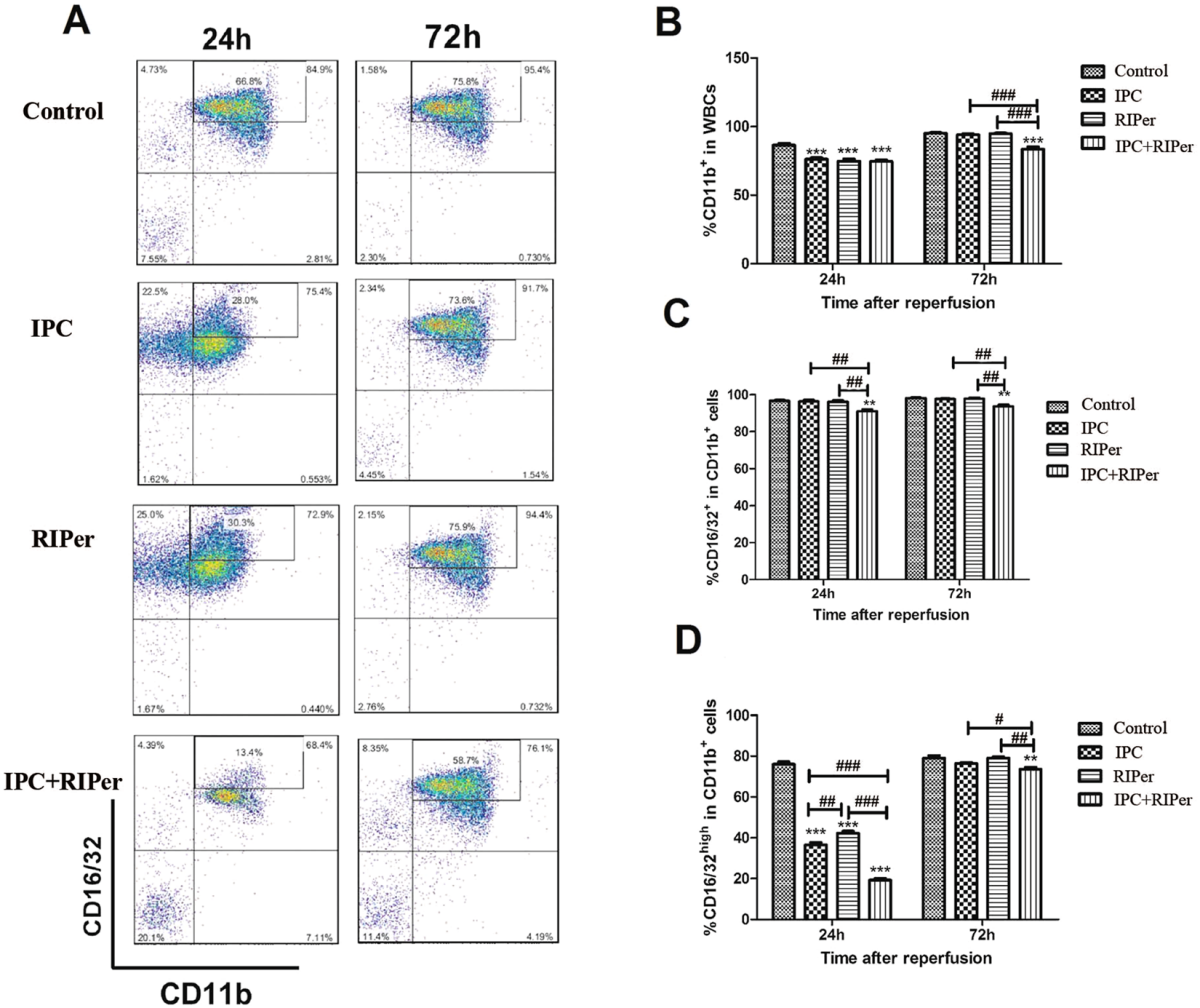
Effect of different maneuvers on the activation of myeloid cells. (**A**) Representative flow cytometry plot of WBCs stained with anti-CD11b and anti-CD16/32 in control, IPC, RIPer and IPC + RIPer group. (**B**) Percentages of CD11b+ cells in WBCs. (**C**,**D**) Percentages of CD16/32+ cells and CD16/32high in CD11b+ cells. *p < 0.05; **p < 0.005; ***p < 0.0005 vs control group, paired Student t-test. ^#^p < 0.05; ^##^p < 0.005; ^###^p < 0.0005 vs different conditioning method, one-way ANOVA and Bonferroni post-hoc test.

In addition to white blood cell number, significant increases in percentages of CD11b^+^ cells among white blood cells, CD16/32^+^ cells among CD11b^+^ cells, and CD16/32^high^ cells among CD11b^+^ cells were detected by flow cytometric analysis. At 24 h after reperfusion, conditioning methods significantly reduced the activation of innate immune cells. In addition, inhibitory effect of IPC was stronger than that of RIPer. At 72 h, although single treatment didn’t have any effect on cell proportions, combined protocol significantly inhibited activation of innate immune system, indicating an enhanced effect (Fig. 3).

**Figure 4:**
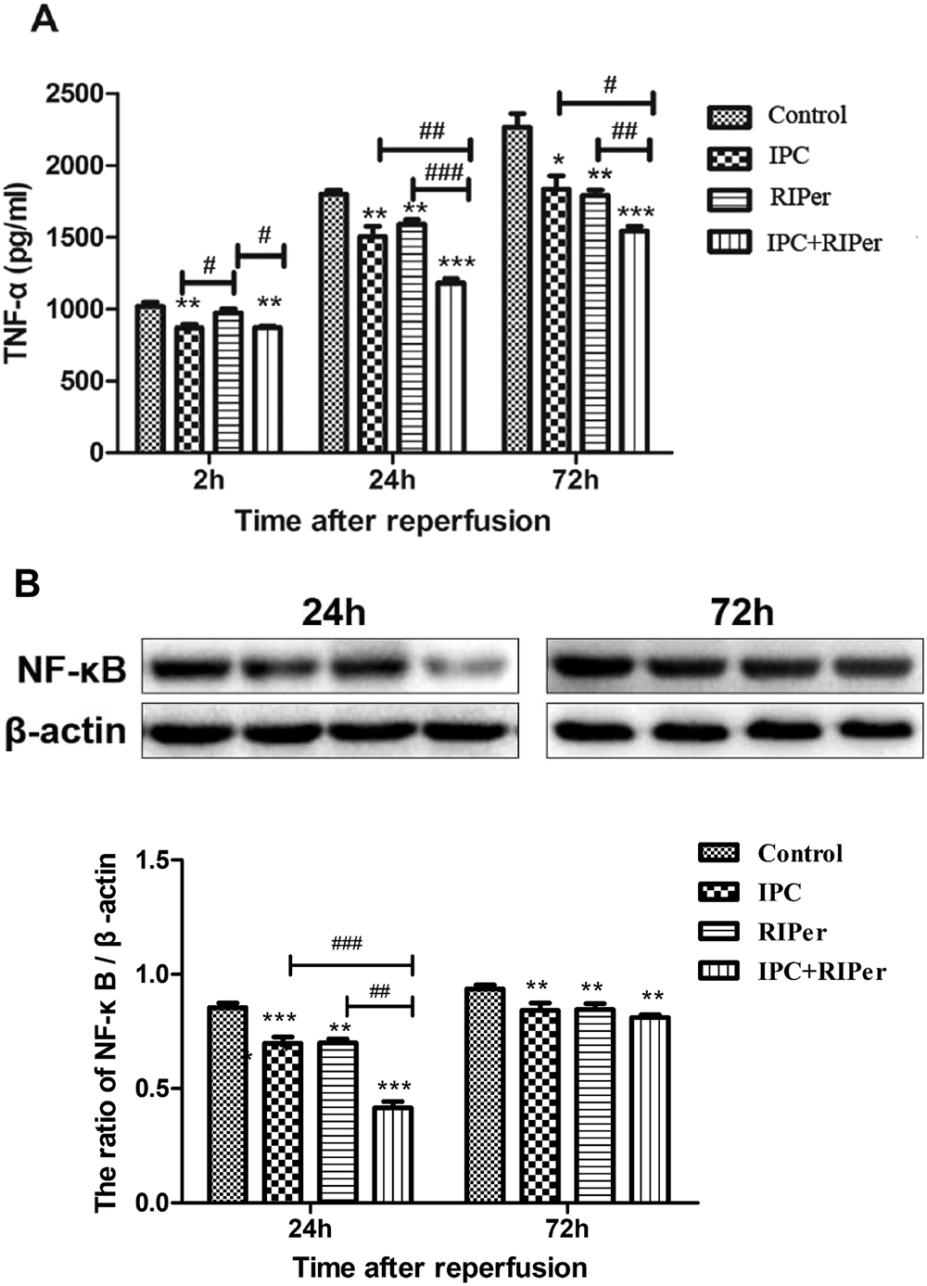
Effect of different maneuvers on the level of serum TNF-α and NF-κB p65 expression. The protection involved inhibitory effect on the release of TNF-α (**A**) and expression of NF-κB p65 (**B**) respectively. *p < 0.05; **p < 0.005; ***p < 0.0005 vs control group, paired Student t-test. ^#^p < 0.05; ^##^p < 0.005; ^###^p < 0.0005 vs different conditioning method, one-way ANOVA and Bonferroni post-hoc test. Full-length gels and the croppings used in this figure are presented in Supplementary Fig. S1.

There was a statistical difference in TNF-α levels between IPC and RIPer groups at 2 h after reperfusion, indicating that RIPer exerted a weaker inhibitory effect on production and release of TNF-α. At the other two time points, the data showed significantly lower levels of TNF-α in the combined treatment group (Fig. 4A).

Compared with control group, each treatment group showed significantly lower expression levels of NF-κB p65 at 24 h and 72 h. IPC + RIPer group showed an additive effect on inhibiting the expression of NF-κB p65, mainly during the early postoperative period (Fig. 4B).

### Effect of the conditioning methods on oxidative stress

**Figure 5:**
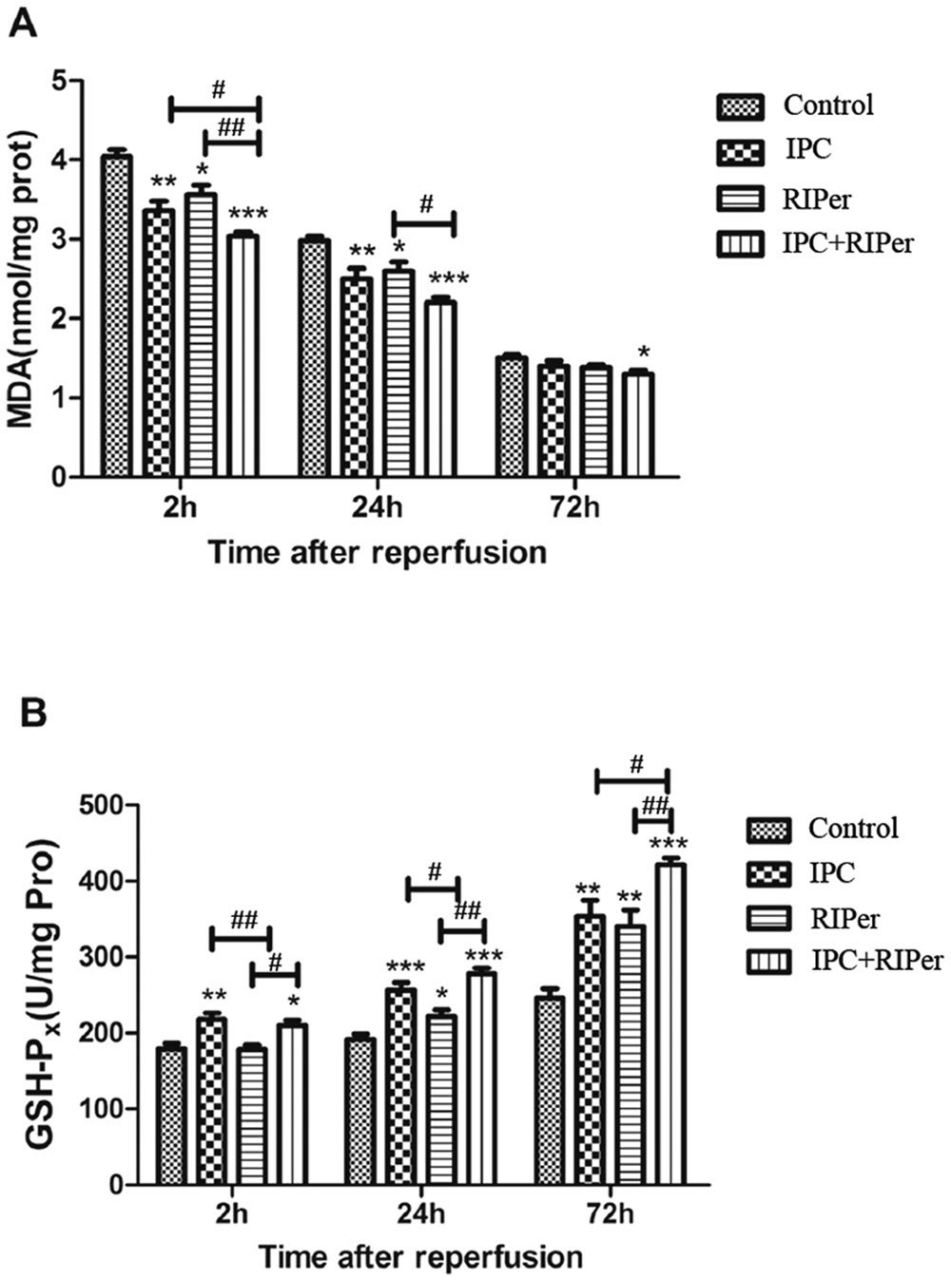
Effect of different maneuvers on the oxidative stress. The antioxidation included inhibitory effect on production of MDA (**A**) and positive impact on level of GSH-Px (**B**). *p < 0.05; **p < 0.005; ***p < 0.0005 vs control group, paired Student t-test. ^#^p < 0.05; ^##^p < 0.005; ^###^p < 0.0005 vs different conditioning method, one-way ANOVA and Bonferroni post-hoc test.

The contents of MDA and GSH-Px showed decreasing and increasing trends respectively. Compared with control group, MDA and GSH-Px content maintained at a lower and higher level respectively in conditioning groups. There was a higher level of GSH-Px in IPC group than RIPer group, but not for MDA content. Significant differences were detected in MDA and GSH-Px levels at 24 h and 72 h respectively, between combined treatment and each single treatment group (Fig. 5).

### Effect of the conditioning methods on liver microcirculation

**Figure 6:**
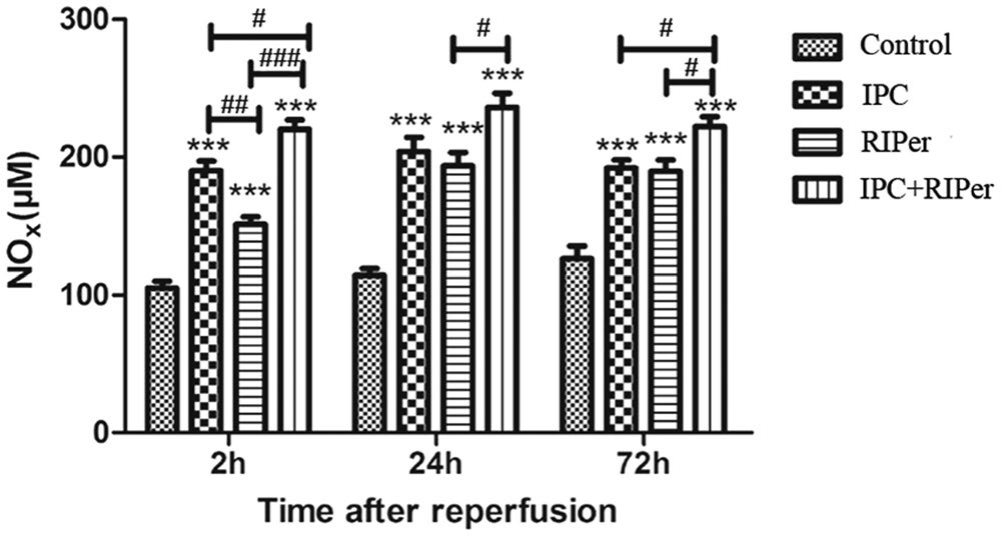
Effect of different maneuvers on liver microcirculation. NOx was chosen to reflect the perfusion quality of microcirculation. *p < 0.05; **p < 0.005; ***p < 0.0005 vs control group, paired Student t-test. ^#^p < 0.05; ^##^p < 0.005; ^###^p < 0.0005 vs different conditioning method, one-way ANOVA and Bonferroni post-hoc test.

Compared with treatment groups, NOx content maintained at a lower level in control group. At 2 h after reperfusion, IPC had a strong positive effect on the generation of NOx. The higher NOx level in IPC + RIPer group demonstrated that combined conditioning provided an enhanced effect (Fig. 6).

### Effect of the conditioning methods on hepatocyte apoptosis

**Figure 7:**
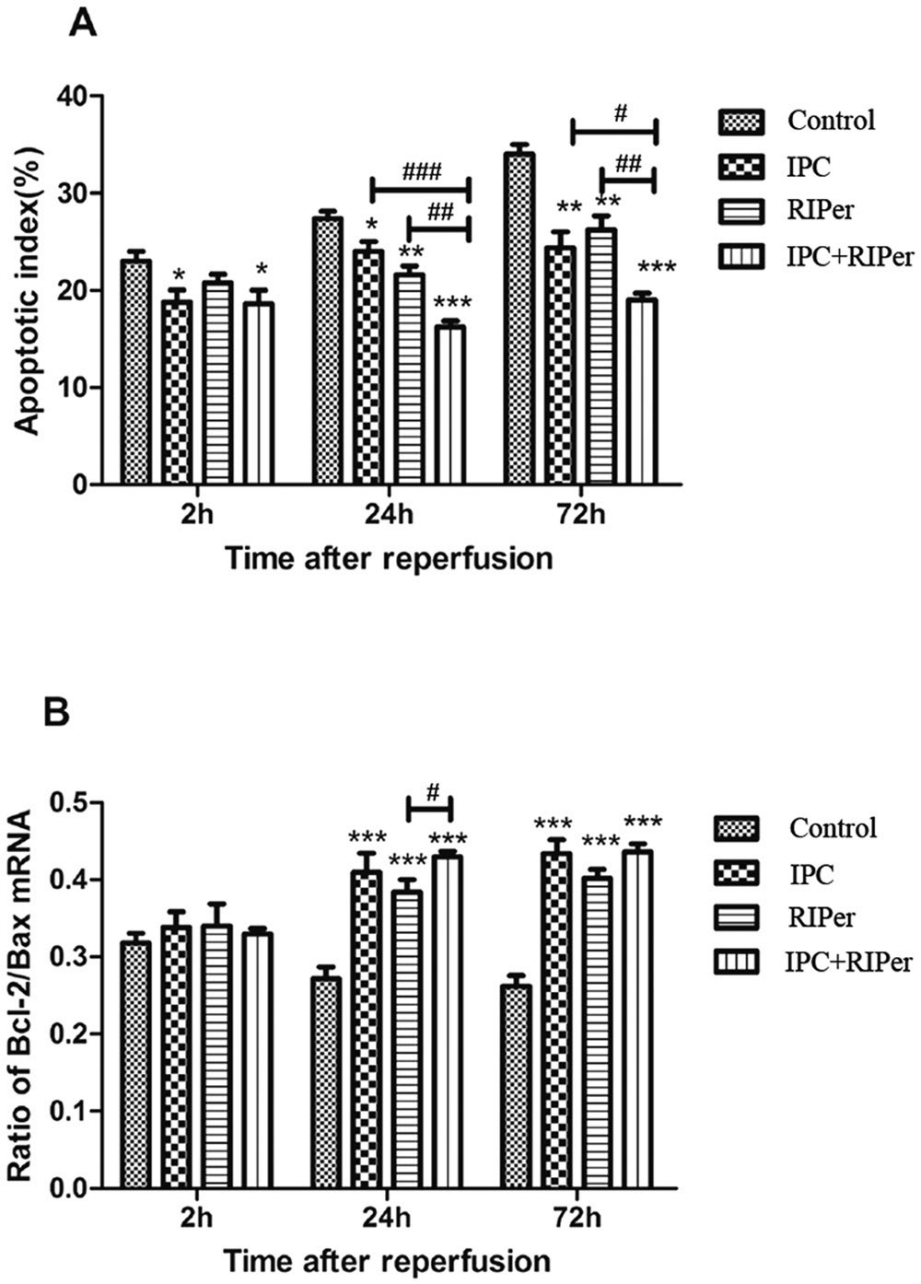
Effect of different maneuvers on hepatocyte apoptosis. The anti-apoptosis effect was reflected in reduced apoptotic index (**A**) and improved Bcl-2/Bax mRNA ratios (**B**). Apoptotic index was determined as the percentage of apoptotic cells per hepatic cell population in five random fields at x400 magnification. *p < 0.05; **p < 0.005; ***p < 0.0005 vs control group, paired Student t-test. ^#^p < 0.05; ^##^p < 0.005; ^###^p < 0.0005 vs different conditioning method, one-way ANOVA and Bonferroni post-hoc test.

Compared with control group, apoptotic indexes and Bcl-2/Bax mRNA ratios maintained at a lower and higher level respectively in conditioning groups. The apoptotic index was significantly reduced in IPC + RIPer group compared with that in single treatment (Fig. 7A). At 24 h and 72 h postoperation, IPC and RIPer groups had equivalent apoptotic indexes and Bcl-2/Bax mRNA ratios (Fig. 7B). These results indicated that combined treatment provided stronger anti-apoptotic effects than single treatment.

## Discussion

Nowadays, IRI remains an inevitable obstacle that could trigger a series of problems after liver transplantation. In present study, although IPC, RIPer, and IPC + RIPer did not affect long-term graft survival, these protocols improved tolerance to liver IRI after liver transplantation. To the best of our knowledge, this is the first study to report that the combination of IPC and RIPer provides further protection against IRI than either alone using a MOLT model. In addition, our data revealed that the protective efficacy of IPC was stronger than that of RIPer in the early phase (2 h postoperation), but there was no difference in the late phase (24 h and 72 h postoperation). The protective effects of the conditioning methods were verified to be associated with antioxidation, anti-apoptosis, modulation of microcirculation disturbance, and inhibition of innate immune responses. Based on the fact that clinical practice of either IPC or RIPer obtained controversial benefit^25–29^, this study provided a new clinically feasible maneuver that may be elicited in patients undergoing liver transplantation to obtain a more satisfactory outcome through reducing complication, cost and hospital stay.

In our opinion, there are mainly three reasons accounting for the fact that the three kinds of conditioning methods have no effect on survival rate. First, after lots of practice, our research center has successfully established the mouse orthotopic liver transplantation model reliably and reproducibly. The deaths mainly concentrated in the first postoperative week were caused from technical causes, not IRI. Second, different from human liver transplantation nowadays, except for artificially prolonged cold ischemic time, neither the donor or the recipient mouse has underlying diseases or related complications of end-stage liver disease. Finally, the liver owns strong self-healing, regeneration and compensatory capacity. According to our preliminary experiment, the cold ischemia time we set could lead to severe but reversible liver injury, which wouldn’t threaten the life.

Hepatic preservation/reperfusion injury is mainly associated with detachment of sinusoidal endothelial cells, activation of liver macrophages, induction of innate immune response, oxidative stress, and ultimately hepatocyte apoptosis^30^. By in-depth exploration of the related mechanisms, numerous preventive and therapeutic countermeasures have been developed to not only improve graft survivability and liver functions, but also increase grafts available to some extent^31^. However, with the increasing trend in utilization of grafts from extended criteria, strategies need to be developed or innovated to enhance the benefit in minimizing the more serious IRI. According to the time and mechanism, protective effect of IPC against hepatic IRI is divided into two time windows. The first takes place immediately and lasts for 2–4 h. The mechanism mainly includes promoting the synthesis of adenosine, increasing the hepatic energy reserve, and inhibiting neutrophil adhesion. The protective effect of the other is not as high as the former, which starts at 12–24 h and lasts for 2–3 days, and depends on expressing new related proteins and altering gene expression levels^32^. IPC was applied in clinical liver transplantation for the first time by Koneru *et al*.^33^. When the period of preconditioning was extended to 10 min, IPC prompted the release of anti-inflammatory cytokine and reduced the risk of early rejection^25^. Recently, a randomized double blind clinical study demonstrated that 10 min of IPC does not provide an evident clinical benefit^26^. One hypothesis proposed to account for these observations is that the protective effect of IPC alone is too low for a clinical manifestation, and the combination of IPC and other procedures might provide stronger protective efficacy for human liver transplantation.

Until now, understanding of the RIPer had lagged behind IPC. In addition, exploration of the former usually refers to IPC and RIPC. Many signaling mediators, including G protein-coupled receptors (adenosine, opioid, bradykinin, endocannabinoid, and angiotensin receptors), stromal cell derived factor-1α, protein kinase C, reactive oxygen species, nitric oxide, TNF-α, calcitonin-generated peptide, Akt, signal transducer and activator of transcription 5, were found in previous studies^34–36^, the data we collected was consistent with that. Otherwise, the additive protection provided by combination of IPC and RIPer was most likely explained by the fact that numerous same signaling molecules, terminal effectors and crossed signal path existed in these two kinds of stimulus, which lead to a signaling cascade. We also put forward a hypothesis that ischemic liver has a memory function to enhance the response to signaling mediators produced again, just like immunological memory. On the other hand, our data showed that in the early phase (2 h) after liver transplantation, the protective efficacy of RIPer is less than IPC, which supported the phenomenon found in a rat RIC model from the aspect of oxidative stress^37^. However, the blocking of the neural pathway caused from denervation of the graft could not be ruled out. Interestingly enough, in the late phase (24 h and 72 h), the detected index can be compared to each other. This phenomenon supported to the hypothesis that RIPer also has two protective time windows similar to IPC, and strongly emphasized RIPer’s advantage over IPC in the second time window. What’s more, the result provided us a hint that RIPer’s mechanism might be partly or totally different from IPC.

In 2011, a study using the rat model of myocardial infarction demonstrated that a single early episode of RIPer could reduce the infarct size^38^. Because of the maneuverability, this strategy has been recently translated into the clinical setting. In 2014, a clinical study showed that either RIPer or IPost reduces the infarct size in ST-elevation myocardial infarction patients. However, combination of the two protocols did not lead to a further decrease in infarct size^27^. In 2015, a different conclusion suggested that combined treatment provides an enhanced effect on myocardial salvage compared with IPost alone^28^. However, in the same year, it was demonstrated that the combination of RIPer and remote IPost did not reinforce the protection against myocardial IRI with a rat model, compared with either treatment alone^29^. Numerous experimental studies have confirmed hepatoprotection of RIPer against warm IRI^39,40^. However, studies of applying RIPer stimulus to liver transplantation are severely lacking, except for reports by Zheng *et al*.^19,20^. Due to inadequate blood occlusion, limb paralysis, and overpressure on both muscles and nerves because of the thinner legs of mice and unstable elasticity of a tourniquet, we chose direct clamping of the femoral vessels instead of using a tourniquet 21. In accordance with the results from Zheng *et al*. using the same RIPer strategy but with a tourniquet in rat liver transplantation, the RIPer stimulus we described could improve prognosis in the early phase after mouse liver transplantation.

In conclusion, this study provided us a fact that combined IPC and RIPer could enhance the protection against IRI and an assumption that some new or different mechanisms existed in RIPer method. No doubt, due to the species specificity and limited mice we used, additional studies in large animals or clinical trials is still needed to verify this phenomenon, and deep exploration and confirmation of new target molecules is also needed to clarify the specific mechanism in the future research.

## Electronic supplementary material

Supplemental Figure S1
