# Supplementary material for: Impact of combined ischemic preconditioning and remote ischemic perconditioning on ischemia-reperfusion injury after liver transplantation
Source: Sci Rep. 2018 Dec 19;8:17979. doi: 10.1038/s41598-018-36365-5 (PMC6299280; doi:10.1038/s41598-018-36365-5)

Impact of combined ischemic preconditioning and remote ischemic perconditioning on ischemia-reperfusion injury after liver transplantation

Ding-yang Li[a], Wen-tao Liu [a], Guang-yi Wang [b], Xiao-ju Shi [b]*

[a] Department of Hepatobiliary & Pancreatic Surgery, The First Affiliated Hospital of Zhengzhou University, Zhengzhou 450000, Henan Province, China

[b] Department of Hepatobiliary & Pancreatic Surgery, the First Norman Bethune Hospital Affiliated to Jilin University, Changchun 130021, Jilin Province, China

* **Corresponding author:** Telephone: +86-13603981433 Fax number: 0371-66279006

Email： Ding-yang Li:740086584@qq.com

Wen-tao Liu: 1083931769@qq.com

Guang-yi Wang: aowang6174@yeah.net

Xiao-ju Shi: 978362967@qq.com

Supplemental Figure S1. Uncropped image for NF-κB p65 and β-actin for Figure 4.

The blue boxes show the lanes displayed in Figure 4.

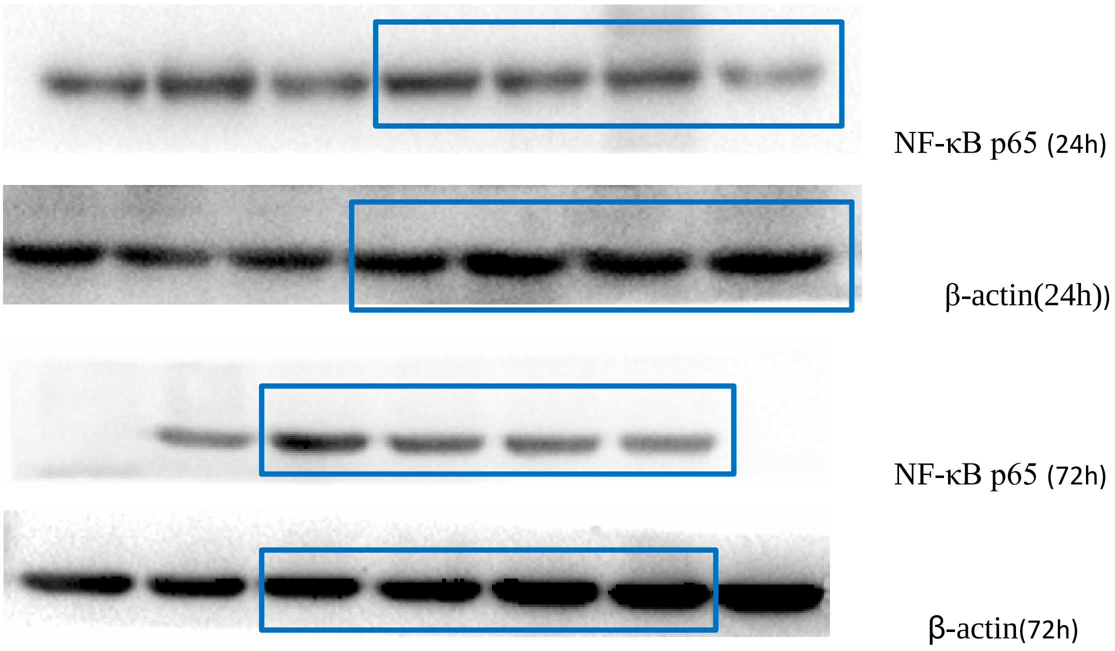

Supplement: Supplementary file 1 — Supplemental Figure S1 [file 41598_2018_36365_MOESM1_ESM.pdf]
